# Engaging people with lived experience to co-design a community-led physical activity and diabetes prevention intervention for people experiencing homelessness

**DOI:** 10.3389/fspor.2026.1764108

**Published:** 2026-06-02

**Authors:** Hayley Cornett, Terry Saunders-Smith, Sarina Aryal, Anna Whaley, Elijah Marfo, Roland Booth, David J. T. Campbell

**Affiliations:** 1 Department of Medicine, University of Calgary, Cumming School of Medicine, Calgary, AB, Canada; 2 Calgary Diabetes Advocacy Committee (CDAC), Calgary, AB, Canada; 3 Department of Community Health Sciences Medicine, University of Calgary, Cumming School of Medicine, Calgary, AB, Canada; 4 Department of Cardiac Sciences, University of Calgary, Cumming School of Medicine, Calgary, AB, Canada

**Keywords:** co-design, community-based participatory research, diabetes mellitus, diabetes prevention, health equity, people with lived experience of homelessness (PWLEH), physical activity intervention

## Abstract

People with lived experience of homelessness (PWLEH) experience disproportionate challenges to preventing diabetes, including access to opportunities for physical activity. In partnership with the Calgary Diabetes Advocacy Committee (CDAC), we co-designed and piloted a low-barrier, community-led Physical Activity Day to promote movement, wellness, and connection among PWLEH. The event featured five stations, stretching and yoga, cardio and steps, strength, balance, and diabetes and nutrition education. The activities incorporated simple equipment, modifiable exercises, and peer-informed staff. Thirteen participants completed all stations and provided pre-event surveys, and eleven participants provided post-event survey data, reporting high satisfaction, feelings of safety and inclusion, and increased motivation to be more active than baseline. Qualitative feedback emphasized enjoyment, social connection, and supportive staff. Limited pedometer data confirmed engagement in the movement-based intervention. Findings demonstrate that a co-designed physical activity event may be feasible, acceptable, and capable of fostering physical and mental health benefits for PWLEH, supporting potential for future scalable community-based interventions.

## 1 Introduction

Physical activity is widely recognized as an established, effective diabetes prevention strategy (1–4). Research consistently shows that individuals who engage in regular movement have lower rates of developing diabetes than those who are minimally active or sedentary (5–9). A Finnish Diabetes Prevention Study (DPS) has illustrated that the risk of diabetes can be reduced by as much as 58% from changes in health behaviours, involving a reduction in the intake of total and saturated fat and an increased intake of fiber, and increased physical activity levels (10). Additionally, a study among individuals with markers of dysglycemia found that health behaviour changes decreased diabetes incidence by 58% compared to placebo, a greater reduction than conferred by metformin, which decreased the diabetes incidence by 31% (5). Long-term follow-up research shows that changes in health behaviours and an increase in physical activity resulted in a reduction in the incidence of diabetes years after the intervention ended. Specifically, three years after the intervention ended, the risk reduction was maintained at 36% demonstrating that the intervention effects attenuate slightly but remain intact (11). Beyond metabolic factors (12), physical activity can also help improve health in a variety of other ways, such as decreases in excess body weight (12, 13), improved cardiovascular health (14, 15), and reduced inflammation (16).

Co-design and community-based participatory research (CBPR) approaches are increasingly recognized as essential for developing interventions that are acceptable, feasible, and contextually relevant, particularly among populations that face social and structural disadvantage (17–20). These approaches prioritize lived experience and ensure that interventions are grounded in the realities of the populations they aim to serve, drive more equitable health outcomes, and avoid reinforcing existing inequities (20, 21). By actively involving community members in all aspects of the project, from priority setting to implementation, co-design creates trust, enhances relevance, and improves engagement (22). This is particularly important for people with lived experience of homelessness (PWLEH), whose complex social and environmental barriers are often insufficiently addressed by existing interventions (23). Consequently, co-designed approaches between researchers and PWLEH offer a promising method for developing equitable, low-barrier health interventions that are applicable to the lived realities and needs of PWLEH.

PWLEH face a higher prevalence of cardiometabolic health issues, when compared to the general housed population (24, 25). They also encounter multiple barriers that make managing their health far more difficult (26–28). Diabetes management is particularly challenging in this population due to unstable housing (27), competing priorities for survival (29), and social stigma (26). Moreover, difficulty accessing healthcare services (28), lack of access to nutritious diabetes friendly food (27, 28), and the effects of homelessness upon mental and emotional well-being (29) pose barriers to diabetes self-management. These complex factors have a detrimental effect on glycemia levels (29–31), resulting in higher rates of diabetes-related complications (32), emergency department and acute care use (32, 33), and ultimately higher mortality rates (34). In fact, one analysis found an association between better housing conditions and improved glycemic parameters (35). Consequently, prioritizing accessible and equitable diabetes prevention efforts, including increased ability to engage in physical activity, is crucial to reducing diabetes risk and complications among PWLEH.

The myriad barriers and challenges experienced by PWLEH also affect their ability to engage in regular physical activity (36, 37). The unstable nature of experiencing homelessness and lack of funds are common barriers to participating in physical activity (37). Physical injury (36), mental health challenges, and addictions have been noted as barriers to participating in physical activity among residents of a homeless shelter (38). Lack of accessible spaces or services for exercising (29) as well as stigma and discrimination encountered at mainstream locations pose barriers to participation in physical activity (39). Moreover, a study examining self-care among PWLEH found that morbidity, disability, and lack of motivation limits engagement in physical activity (40). Competing daily life priorities such as finding food often takes precedence over spending time completing structured exercise (40). Research shows that successful interventions must be flexible, adaptable, and supportive of social connection to address the unique needs and priorities of this population (41).

PWLEH face persistent environmental and psychological stressors, including housing stability, food insecurity, stigma, and trauma, which contribute to poorer health outcomes such as diabetes and complications (24–32). Chronic stress produces an “allostatic load,” described as dysregulation of neuroendocrine and metabolic systems, promoting insulin resistance and affecting glucose metabolism (42), which are two key mechanisms in type 2 diabetes development (43). Psychological trauma and stress disorders are prevalent among PWLEH (44), which further elevate cardiometabolic disorders (45–48). Together, these stressors add to the systemic barriers to create a disproportionate burden observed among PWLEH (48). Physical activity may help mitigate both physiological and psychological stress by improving mood, supporting well-being, and improving health outcomes (49–51). Specifically, physical activity has been associated with reductions in perceived stress (52), anxiety (53), and symptoms of depression (54), suggesting that interventions focused on exercise may support physical and psychological well-being in populations experiencing adversity. While exercise is recognized as a physiological stressor, it functions as eustress, the positive form of stress that enhances resilience, promoting beneficial adaptations (55). Accessible, low-barrier opportunities for movement and social engagement may therefore be particularly important for supporting overall health among PWLEH. In 2024, our community-based participatory research group; the Calgary Diabetes Advocacy Committee (CDAC), which is composed of individuals with relevant lived experience, completed a priority setting process to identify what projects we would work on in the coming years. Using the Delphi-Community Prioritization Index methodology (56), CDAC members brainstormed, discussed, and ranked collective priorities according to importance and feasibility. Among eight possible focus areas, including access to medicine, food, shelter, education, and awareness, “promoting healthy lifestyles” among this population was ranked as the top priority. This reflects the members' shared belief that accessible, community-based physical activity and wellness initiatives are urgently needed for diabetes prevention.

## 2 Methods

### 2.1 Calgary Diabetes Advocacy Committee (CDAC)

The CDAC is a CBPR group consisting of academic researchers from the University of Calgary and community members with lived experience of homelessness and diabetes who do not have formal research training. The aim of the group is to improve experiences and outcomes for their peers by designing, piloting, and evaluating interventions that address the multitude of barriers faced by their community. CDAC has led initiatives such as the development of the Diabetes Mobile Clinic (DMC) in partnership with The Alex Community Health Centre (57), and the production of short films Low and Low Priority to capture the lived experiences of healthcare workers, shelter staff, and people with lived experience of navigating diabetes care in the under-resourced shelter and healthcare environment (58). By making lived experience the center of research and implementation, CDAC demonstrates how peer-led community research and advocacy projects can produce feasible, impactful solutions and outcomes that might otherwise be overlooked in conventional research.

### 2.2 Design of the activities

The Physical Activity Day was co-designed through iterative consultation and planning with CDAC members, researchers, registered yoga and fitness instructor, and a registered dietitian. Guided by a participatory approach, CDAC met to discuss and identify feasible, modifiable exercises to promote physical activity in an inviting, engaging, and accessible manner.

The group decided to focus on five key areas: (1) cardio and step-based activities, (2) balance activities, (3) strength exercises, (4) stretching and yoga, and (5) diabetes and nutrition education. Feedback from CDAC members directly shaped the selection of activities, event format, and the incentives and refreshments offered: complementary diabetes testing (point-of-care A1C), healthy snacks, water, and sugar free electrolyte replacement beverages. The participatory and iterative design approach to come up with the five physical activity components ensured that the intervention reflected participant preferences and needs, reduced participation barriers, and aligned with the broader goal of promoting diabetes awareness and prevention.

### 2.3 Description of the intervention

The Physical Activity Day consisted of a single day, outdoor, community-based circuit-style event. Participants were recruited through word-of-mouth from our CDAC members, posters that were put up near the event, and community outreach (see Section 2.4). Upon arrival, participants were provided with informed consent and study information. If consent was obtained, participants were provided with a pedometer and an “activity passport” to track progress. Instructions were provided on how and where to wear the pedometer. Participants were also provided with the option to participate in the event without data collection. When participants were ready to start, they were invited to a station with availability and rotated through the five activities. Each station was carried out by trained staff who provided instruction, demonstrations, and modifications for different activity and experience levels. The cardio and steps station consisted of walking-based activities and step accumulation, strength station involved bodyweight or resistance-based exercises, the balance station involved coordination exercises, the stretching and yoga station included guided low-intensity mobility and flexibility movements, and the education station provided brief education on diabetes prevention and nutrition.

The event staff trained participants in exercises as well as modifiable options for different skill and mobility levels. The event blended exercise, games, social engagement, education, and incentives that included complementary A1C testing and a chance to win a gift card.” The intervention included general diabetes education; however, the primary focus was on promoting physical activity as a diabetes prevention and risk reduction strategy.

The order of the stations was not particular, excluding the education station, which was completed last. After this station was completed, participants had the option to participate in complementary A1C testing, and the opportunity to pick a variety of healthy snacks to take home.

The intervention was designed to be low-barrier, requiring no prior experience, no specialized equipment, and minimal time commitment, with the goal of maximizing accessibility for PWLEH.

### 2.4 Recruitment

Participants were recruited using a combination of community-based outreach prior to the event and on-site engagement on the day of the intervention. In the 60 days leading up to the event, CDAC members promoted the Physical Activity Day through word-of-mouth and distributing posters at community organizations serving individuals experiencing homelessness, including shelters and outreach centres.

On the day of the event, both passive and active recruitment strategies were used. Passive recruitment involved individuals independently approaching the event booth, where study information and consent procedures were completed. Active recruitment involved CDAC members approaching individuals in the surrounding area, providing a brief description of the event, and inviting them to participate.

### 2.5 Data collection and analysis

Pre-intervention physical activity levels were assessed using the Godin-Leisure Time Exercise Questionnaire (GLTEQ) (59). The survey collects information on habitual frequency and intensity of exercise in a typical week, which are multiplied by activity levels to yield a leisure activity measure. Additionally, demographic data and general health information were collected to provide context for findings. Post-intervention questions were administered to gather feedback on participants' experiences, perceived effectiveness of the event, and overall satisfaction with their participation. Step counts were collected to assess the event's effectiveness in promoting movement. Data were intended to be analyzed using descriptive measures only, as we did not anticipate having sufficient sample size to permit any inferential statistical analysis.

### 2.6 Inclusion and exclusion criteria

All participants were welcomed into this pilot study, and no exclusion criteria were applied.

## 3 Results

### 3.1 Participant demographics

Thirteen individuals participated in the Physical Activity Day. Participants ranged in age from early adulthood to seniors, born between 1940 and 2005. Two gender identities were represented including men (61.5%) and women (38.5%). Participants identified with a range of racial and ethnic backgrounds, including Black-African, Asian-South, Asian-East, Indo-Caribbean, and Caucasian.

Housing situations varied among participants, including individuals residing in shelters, living “place-to-place,” renters, and homeowners. The duration of homelessness varied up to 19 months, while others declined to answer. Several participants reported chronic health conditions, including diabetes (*n* = 2), high blood pressure (*n* = 2), and heart disease (*n* = 1). Additional baseline characteristics are summarized in Table 1.

**Table 1:** Baseline characteristics of participants (*n* = 13).

Characteristics	*n* (%) or Range
Year of Birth	1940–2005
Gender	
Male	8 (61.5%)
Female	5 (38.5%)
Racial/Ethnic Identity	
Black/African	2 (15.4%)
Asian (South) or Indo-Caribbean	6 (46.2%)
Asian (East)	2 (15.4%)
Caucasian	3 (23.0%)
Housing Status	
Shelter	2 (15.4%)
Unstable “place-to-place”	2 (15.4%)
Renter	4 (30.8%)
Homeowner	4 (30.8%)
Not Answered	1 (7.6%)
Chronic Health Conditions	
Diabetes	2 (15.4%)
Hypertension	2 (15.4%)
Heart Disease	1 (7.6)
No health conditions listed	8 (61.5)
Habitual Physical Activity Measure	
GLTEQ Score	Range 20–91

### 3.2 Baseline health and physical activity (pre-intervention survey findings)

Baseline self-reported health and functioning varied across participants. Responses to general health questions indicated a range of perceived quality of life, physical health, mental health, and perceived ability to carry out daily activities. The Majority of participants reported fair to good functioning across the Likert scale, however, responses varied across the spectrum (Table 2).

**Table 2:** Participants' responses regarding their general health and functioning (*n* = 13).

Baseline Health and Physical Activity Questions	Poor *n* (%)	Fair *n* (%)	Good *n* (%)	Very Good *n* (%)	Excellent *n* (%)
In general, how would you rate your quality of life?	2 (15%)		3 (23%)	4 (31%)	2 (15%)
In general, how would you rate your physical health?	1 (8%)	2 (15%)	3 (23%)	3 (23%)	2 (15%)
In general, how would you rate your mental health?	0	3	3 (23%)	3 (23%)	2 (15%)
In general, how would you rate your ability to carry out daily activities?	1 (8%)	2 (15%)	2 (15%)	2 (15%)	4 (31%)

Baseline activity levels, measured by the Godin Leisure-Time Exercise Questionnaire (GLTEQ), ranged from 20 to 91. Based on scoring categories, five participants were classified as active and one participant as moderately active, while several respondents declined to answer.

### 3.3 Intervention engagement

Engagement with the event was high, all thirteen participants completed the stations and the pre- survey, and eleven participants completed the post- survey. Pedometer data were available for five participants. Step counts recorded during the event ranged from 168 to 1,163.

### 3.4 Participant experience and perceived impact

Participants reported overwhelmingly positive experiences. Across all Likert-scale items, all answers were either “agree” and “strongly agree” (Table 3).

**Table 3:** Participants' responses regarding feedback from the Physical Activity Day (*n* = 11).

Post-Intervention Questions	Agree *n* (%)	Strongly Agree *n* (%)
I felt included and supported at the activity day.	3 (27%)	8 (73%)
I felt safe at today's event.	3 (27%)	8 (73%)
I had fun participating in the activity day.	2 (18%)	9 (82%)
This event encouraged me to move more than I usually do.	4 (36%)	7 (64%)
I would like to come to an event like this again.	5 (45%)	6 (55%)
I made meaningful social connections during this event.	4 (36%)	7 (64%)

### 3.5 Qualitative feedback

Open-ended feedback from participants further illustrates the positive reception of the event. Participants described the event as enjoyable and expressed interest in attending similar activities in the future. Social interaction, the supportive atmosphere, and learning new information related to diet and diabetes education were also reported as highlights (Table 4).

**Table 4:** Themes and quotes from qualitative feedback from the post-intervention survey.

Theme	Quote
Enjoyment and Engagement	“[I liked] everything [about today's event]. Nice. Fun. I would like to do it again”
Social Connection	“I enjoyed the physical activity and talking to people.”
Support and Safety	“It was good to attend and get support. It was very safe activities.”
Educational Value	“I learn more information about the diet plan and learning diabetes education.”
Positive Staff Interactions	“The staff/people were nice.”
Holistic Benefit	“All of today's event was good and well thought out.”

## 4 Discussion

Physical activity is associated with the potential for to improve blood glucose levels (12), reduce cardiovascular risk (14, 15) and enhance mental health (29). In addition to metabolic benefits, physical activity may help mitigate the chronic environmental and psychological stress experienced by PWLEH, which is increasingly recognized as a contributor to cardiometabolic disease (60). For individuals experiencing homelessness, opportunities for accessible and inclusive physical activity may also contribute to improved psychological well-being and stress regulation (51, 61). Given the increased prevalence and disproportionate burden of diabetes among PWLEH and the barriers faced while managing their condition with conventional care, physical activity represents a critical, low-cost, and modifiable behaviour for improving outcomes (4). Consequently, there is a need for interventions to improve health outcomes in PWLEH, but there remains a gap in the literature on effectiveness of physical activity interventions (62). The CDAC Physical Activity Day was feasible, well-received, and successful in engaging individuals with lived experience of homelessness in low-barrier, community-designed physical activity. Participants reported high satisfaction, strong perceptions of safety and inclusion, meaningful social connection, and motivation to increase physical activity beyond their usual levels (Tables 3, 4). Pre-intervention data highlight substantial variability and barriers faced by this population, underscoring the importance of accessible, community-led interventions (Table 2).

The CDAC Physical Activity Day was successful among the thirteen participants, demonstrating that an inclusive, low-barrier, peer-designed intervention can be feasible and acceptable for PWLEH. Despite the barriers to structured physical activity typically faced by this population, all participants completed the event, engaged with each activity station, and provided positive feedback. Qualitative feedback highlighted supportive interactions with staff and increased motivation to move more than usual.

The design of the intervention explicitly considered the structural barriers faced by PWLEH such as financial constraints, unstable living conditions, and restricted access to fitness classes or recreational facilities. CDAC members carefully selected activities that required minimal equipment, were adaptable to physical ability and experience, and were deliverable and in an accessible environment. The involvement of community members from CDAC further enhanced relevance and acceptability, which are critical factors when building an intervention for PWLEH (23). Looking at sustainability, this program has potential to continue in different formats (i.e., incorporating basketball tournaments) and environments (i.e., indoor and outdoor). Moreover, this intervention could be adopted into existing programs, organizations, shelters, or outreach programs, particularly if delivered as a recurring, low-cost event. Sustainability would also require funding, community collaboration, and ongoing engagement and planning with PWLEH to ensure continued relevance.

PWLEH are at an increased risk of developing diabetes and related cardiometabolic conditions (24, 25). Once diagnosed, diabetes management is often significantly more challenging due to structural barriers such as unstable housing, limited access to healthcare, and food insecurity (26–28, 63, 64). These factors contribute to poorer glycemic outcomes and higher complication rates (65). Consequently, prevention and proactive risk reduction strategies represent a pragmatic approach to reducing the burden of diabetes among PWLEH. Interventions that encourage and promote accessible and low-barrier options to improve health outcomes (i.e., physical activity) may offer a promising path forward to support prevention of diabetes in this population.

A key strength of this pilot was the participatory co-design with members of CDAC. Planning an event rooted in lived experience created an environment that felt safe, respectful, and culturally relevant to participants, which likely contributed to the satisfaction ratings on the survey and themes of safety and enjoyment. The positive experiences reported by all the participants demonstrate the potential impact of community-led approaches, even when implemented on a small scale. The exercises selected were intentionally simple, easily adaptable, and required minimal equipment, suggesting potential for scalability and suited for resource-limited settings.

The small sample size limits the generalizability of findings. While this intervention was limited to a single-day event, it was conceptualized as a preliminary step toward a recurring, community-led program. As a feasibility-oriented pilot event, enrollment of a small sample size was expected, yet it restricts the interpretation of the results to descriptive trends. Missing pedometer data and using base models of pedometers highlights the practical challenges of real-world data in community-based settings with limited funds, nonetheless, pedometer data suggests that participants successfully engaged in the movement-based intervention. Moreover, attendance could have been affected by the proximity of another event with more prominent signage, demonstrating the sensitivity and unpredictability of interventions like this due to external factors that are beyond the team's ability to control.

Baseline participant characterization was limited to reduce participant burden in this low-barrier setting. This study did not formally measure psychological stress or trauma, which may influence both diabetes risk and engagement in physical activity among PWLEH. Future research should consider incorporating valid measures of stress, trauma history, and mental health to better understand the broader impacts of community-based physical activity interventions. Lastly, future iterations of this intervention should consider undertaking a full-scale qualitative study of the participant perspectives and themes amongst open-ended responses.

Given the challenges associated with diabetes management in this population, prevention-focused, accessible interventions may represent a more feasible strategy. In comparison to larger scale physical activity interventions that rely on gym access, extensive curricula, or medication intervention, the approach we piloted offers an alternative, scalable model based on lived experience and inclusive, low-barrier design. Prior research shows that PWLEH face challenges and barriers to participating in regular physical activity such as stigma and logistical barriers (26, 28, 29). The positive feedback from the attendees possibly suggests that a community-led approach may help mitigate these barriers more than traditional physical activity programs would. In contrast to interventions requiring equipment purchases such as soccer or multi-week commitment in the Finnish DPS study, this event was strategically designed to minimize barriers by requiring no special equipment, transport or previous experience (41, 66).

The feasibility of this intervention is further supported in combination with existing literature on shelter-based studies. One study has found that even brief physical activity interventions have potential to improve diet quality and increase physical activity among residents in homeless shelters, demonstrating that interventions like ours can achieve meaningful engagement (67). Similarly, the Finnish DPS demonstrated that changes in health behaviours are directly associated with a reduction in the incidence of diabetes (66). However, the Physical Activity Day's limited sample size and single session limits the conclusions we can draw from such a pilot. While other trials report positive results with respect to diet quality (67), metabolic factors, and physical activity levels (5, 10), this intervention presents preliminary evidence focused on feasibility and acceptability rather than long term change in metabolic factors or behaviours.

This pilot intervention brings up several points that need to be addressed in the future. It remains unknown if a recurring series of physical activity events would lead to sustained changes in behaviour bringing about increases in physical activity, reduced diabetes risk, or improved well-being. Future research should investigate how a scalable model with larger sample sizes and additional resources for promotion could increase feasibility and acceptability. Moreover, future interventions may have more success if the event is thoroughly and strategically promoted to the appropriate population. Collecting additional data such as heart rate and more detailed baseline participant characteristics may help assess the impact of such events as well. Additional research is required to establish the ideal frequency and duration and if peer-led events can be sustainable and adapted into longer-term community programs.

Future studies should consider including whether participants that are PWLEH are military veterans (68) since there is a higher prevalence of homelessness in this population compared to the general population. Including standardized measures of lifestress or trauma, such as the Life Events Checklist for DSM-5 (69), and assessments of trauma-induced stress severity, such as the PTSD Checklist-5 (PCL-5) (70), would provide important information on the types and intensity of trauma and stress experienced. Collecting these baseline measures could help identify the levels of distress participants are experiencing and inform additional lifestyle interventions. Additionally, continuing pre-post assessments with the addition of these tools may help assess the degree to which physical activity interventions reduce stressors over time.

These limitations aside, our findings suggest that a co-designed, low-barrier, and enjoyable environment is conducive to successfully engaging PWLEH in physical activity. The meaningful social connections mentioned by participants indicate that social factors may be just as important as the physical activity aspect. For a population disproportionately affected by chronic disease and social isolation, even brief instances of movement and connection can support motivation and openness for physical activity and modifiable health behaviours (40, 41). Moreover, this event demonstrates that a simple physical activity and nutrition education intervention can provide a platform and opportunity to provide broader health promotion. For healthcare providers and policymakers, this pilot provides preliminary information on how physical activity can be integrated into community settings that are regularly accessed by PWLEH.

## Data Availability

The raw data supporting the conclusions of this article will be made available by the authors, without undue reservation.
